# Relationship between Alcohol Consumption and Components of the Metabolic Syndrome in Adult Population from Maracaibo City, Venezuela

**DOI:** 10.1155/2015/352547

**Published:** 2015-12-08

**Authors:** Valmore Bermúdez, María Sofía Martínez, Mervin Chávez-Castillo, Luis Carlos Olivar, Jessenia Morillo, José Carlos Mejías, Milagros Rojas, Juan Salazar, Joselyn Rojas, Roberto Añez, Mayela Cabrera

**Affiliations:** Endocrine-Metabolic Research Center, “Dr. Félix Gómez”, Faculty of Medicine, University of Zulia, Maracaibo 4004, Zulia State, Venezuela

## Abstract

*Introduction.* Although the relationships between alcohol and disorders such as cancer and liver disease have been thoroughly researched, its effects on cardiometabolic health remain controversial. Therefore, the objective of this study was to assess the association between alcohol consumption, the Metabolic Syndrome (MS), and its components in our locality.* Materials and Methods.* Descriptive, cross-sectional study with randomized, multistaged sampling, which included 2,230 subjects of both genders. Two previously determined population-specific alcohol consumption pattern classifications were utilized in each gender: daily intake quartiles and conglomerates yielded by cluster analysis. MS was defined according to the 2009 consensus criteria. Association was evaluated through various multiple logistic regression models.* Results.* In univariate analysis (daily intake quartiles), only hypertriacylglyceridemia was associated with alcohol consumption in both genders. In multivariate analysis, daily alcohol intake ≤3.8 g/day was associated with lower risk of hypertriacylglyceridemia in females (OR = 0.29, CI 95%: 0.09–0.86; *p* = 0.03). Among men, subjects consuming 28.41–47.33 g/day had significantly increased risk of MS, hyperglycemia, high blood pressure, hypertriacylglyceridemia, and elevated waist circumference.* Conclusions.* The relationship between drinking, MS, and its components is complex and not directly proportional. Categorization by daily alcohol intake quartiles appears to be the most efficient method for quantitative assessment of alcohol consumption in our region.

## 1. Introduction

Alcohol consumption has become a widely prevalent life style in a multitude of societies and cultures, with approximately 40% of the world's population drinking regularly [1]. This behavior is in continuous expansion despite the numerous deleterious effects it bears on health, with an estimate of 2.5 million premature deaths attributed to inappropriate alcohol intake every year [2].

Although the relationships between chronic alcohol consumption and disorders such as cancer and liver disease have been thoroughly studied [3, 4], its cardiometabolic impact remains controversial. Various reports have found low-to-moderate alcohol intake to significantly reduce cardiovascular mortality [5] and risk of developing Type 2 Diabetes Mellitus [6], supporting a beneficial role for alcohol in this context. Nonetheless, excessive consumption has also been linked to increased risk of obesity [7], dyslipidemia [8], hyperglycemia [9], and hypertension [10], all of which are cardiovascular risk factors and components of the Metabolic Syndrome (MS).

The prevalence of MS varies largely across demographics [11], partly due to the influence of cultural factors autochthonous to each population [12]. In this regard, research assessing the relation between drinking patterns and MS prevalence is scarce in our locality [13]. Thus, the objective of this study was to evaluate the association between alcohol intake and MS and its individual effect on each of its components among adult subjects from Maracaibo City, Venezuela, based on previously determined population-specific alcohol consumption patterns.

## 2. Materials and Methods

### 2.1. Sample Selection

This report is part of the Maracaibo City Metabolic Syndrome Prevalence Study (MMSPS), a cross-sectional study whose purpose is to identify Metabolic Syndrome and cardiovascular risk factors in the adult population of the Maracaibo, the second largest city of Venezuela. The sample (1,986 individuals) was calculated based on estimations of the city's population by our National Institute of Statistics (1,428,043 inhabitants for the year 2007). A total of 244 subjects (12%) were added for oversampling, in order to increase accuracy of the estimates obtained from smaller subgroups from the overall sample, amounting to a total of 2,230 individuals. Maracaibo City is divided in parishes, each of which was proportionally sampled with a multistage cluster method: In the first stage, clusters were represented by sectors from each of the 18 parishes, selecting 4 from each parish by simple randomized sampling. In the second phase, clusters were represented by city blocks within each sector, which were selected by simple randomized sampling using a random number generation tool. From the overall population, 2,026 individuals were selected on the basis of availability of insulin determination. Further details of the sampling process have been previously published elsewhere [14].

### 2.2. Ethical Considerations

All individuals enrolled in the study signed a written informed consent before undergoing physical examination and blood sample collection. All procedures were approved by the Ethics Committee of the Endocrine and Metabolic Diseases Research Center of The University of Zulia, Maracaibo, Venezuela.

### 2.3. Subject Evaluation

Data were collected through completion of a full clinical record carried out by trained personnel, which included interrogation regarding personal and family medical history, with an emphasis on current or past acute or chronic liver disease. Likewise, ethnic origin, educational status, occupational status, tobacco use, and socioeconomic status, according to the Graffar scale modified by Méndez Castellano and de Méndez [15], were also investigated. The Long Form of the International Physical Activity Questionnaire (IPAQ-LF) was used for the evaluation of physical activity; its design allows for the assessment of PA in four domains: work, transportation, leisure, and household activities.

### 2.4. Alcohol Consumption and Drinking Patterns

**Figure 1:**
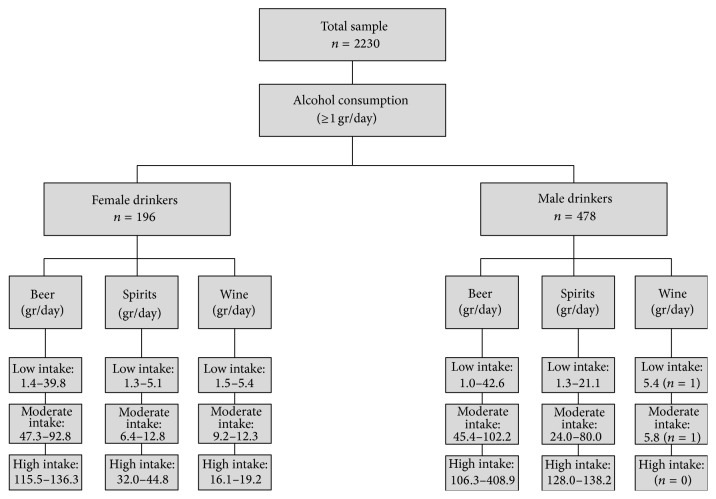
Diagram showing the processing of the sample applying two-staged cluster analysis for categorizing subjects according to gender, type of beverage, and daily alcohol intake. Maracaibo, 2015.

For the assessment of alcohol intake, subjects were asked to estimate the amount of alcoholic drinks they consumed within a month, with the approximate quantity and frequency of daily intake for each type of drink: beer, spirits, and wine and its derivatives. Accounting for the standard content of alcohol grams in each kind of beverage [16], daily intake of alcohol grams was calculated through the formula [17]:

(1) $$\begin{array}{c} \frac{Daily\;\;Consumed\;\;\left(mL\right)\times Degrees\;\;of\;\;Alcohol\times 0.8}{100}, \end{array}$$

where 0.8 is a constant which represents ethanol density in drinks. Based on this estimation, “habitual drinkers” were defined as subjects who consumed ≥1 gram of alcohol daily [18]. These individuals were then categorized by two distinct methods previously described in our report evaluating drinking patterns in Maracaibo city [19]:

1. Gender-specific quartiles of alcohol grams consumed daily (25th percentile, 50th percentile, and 75th percentile).
2. Three gender-specific conglomerate categories obtained from two-staged cluster analysis, which allows separation of patterns into clusters, sets, or groups, according to distance criteria (Figure 1). This technique was used to include quantity and type of alcohol beverage (beer, wine, and spirit drinks); in order to evaluate the quality of the resulting clusters, the cohesion, separation, and silhouette coefficient were calculated.

### 2.5. Evaluation of Blood Pressure

Blood pressure (BP) was taken with subjects sitting down with their feet on the floor following 15 minutes of rest, determined through the auscultatory method with a calibrated mercury sphygmomanometer, identifying Korotkoff's phases I and V as systolic and diastolic BP, respectively. BP was determined 3 times, with 15 minutes in between each take, on two different days.

### 2.6. Anthropometric Assessment

Waist circumference (WC) was measured using calibrated measuring tapes in accordance with the anatomical landmarks proposed by the USA National Institutes of Health protocol [20]. Height was obtained using a calibrated rod, millimeters and centimeters, with the patient barefooted and his/her back facing the wall. Weight was recorded using a digital scale (Tanita, TBF-310 GS Body Composition Analyzer, Tokyo, Japan) with the patient using light clothing and no shoes. Body Mass Index (weight/height^2^) was expressed in kg/m^2^.

### 2.7. Laboratory Analysis

Overnight fasting determination of glucose, total cholesterol, triacylglycerides (TAG), and HDL-C was done with an automated analyzer (Human Gesellschaft für Biochemica und Diagnostica mbH, Germany); the intra-assay variation coefficients for total cholesterol, TAG, and HDL-C were 3%, 5%, and 5%, respectively. LDL-C and VLDL-C levels were calculated applying Friedewald's formula [21] when TAG levels were <400 mg/dL. With TAG levels above this cutoff, LDL-C concentrations were measured through lipoprotein electrophoresis and densitometry with BioRad GS-800 (BioRad).

### 2.8. Diagnosis of Metabolic Syndrome

MS was diagnosed using the criteria from the IDF/AHA/NHLBI-2009 consensus [22], which requires the presence of ≥3 of the following components: (a) low HDL-C, <50 mg/dL in females or <40 mg/dL in males; (b) high TAG, ≥150 mg/dL; (c) elevated WC, ≥80 cm in females or ≥90 cm in males; (d) hyperglycemia, fasting glycemia ≥100 mg/dL, or personal history of DM2 or prescription of hypoglycemic drugs; and (e) high blood pressure, BP ≥130/85 mm/Hg or previously diagnosed hypertension or prescription of antihypertensive drugs.

### 2.9. Statistical Analysis

Qualitative variables were expressed as absolute and relative frequencies and were assessed for associations with Pearson's Chi-squared (*χ* ^2^) test. Qualitative variables were evaluated for distribution normality with Geary's test and were expressed as arithmetic means ± standard deviation. One-way ANOVA was used to evaluate differences between means from ≥3 groups, with post hoc Tukey analysis in cases with statistical significance. Variables with nonnormal distribution, such as daily alcohol intake, were expressed as medians (25th percentile–75th percentile). Various gender-specific multiple logistic regression models were constructed in order to estimate odds ratios (95% CI) for the presence of MS and each of its separate components, adjusting for age groups, ethnic groups, socioeconomic status, educational status, occupational status, family history of hypertension and diabetes, tobacco use, four domains of physical activity, and both categorizations for drinking patterns (quartiles of alcohol grams consumed daily and conglomerates from cluster analysis). Data were analyzed with the Statistical Package for the Social Sciences (SPSS) v.21 for Windows (IBM Inc. Chicago, IL). Results were considered statistically significant when *p* < 0.05.

## 3. Results

### 3.1. Prevalence of Metabolic Syndrome and Alcohol Consumption

A total of 2,230 subjects were studied (52.6% females, *n* = 1,172), with a mean age of 39.3 ± 15.4 years. General characteristics of the population are shown in Table 1.  Table 2 shows the prevalence of MS by gender and quartiles of daily alcohol intake. Among women, MS prevalence increases progressively across quartiles, with the greatest consumption in the fourth quartile (≥28.41 g/day) (42.5%, *n* = 17; *χ* ^2^ = 9.332; *p* = 0.053). In men, the greatest prevalence of MS was in the group consuming 28.41–47.33 g/day, with 55.1% (*n* = 49), with no statistically significant association between these variables.

On the other hand, Table 3 displays the prevalence of MS by gender and drinking pattern conglomerates. In females, MS prevalence appears to increase across categories (low intake: 31.9% versus high intake: 55.6%; *χ* ^2^ = 6.372; *p* = 0.095). In males, MS prevalence was similar in the groups with moderate and high intake (50%; *χ* ^2^ = 3.166; *p* = 0.367).

### 3.2. Components of the Metabolic Syndrome and Alcohol Consumption

When assessing the association between separate MS components and quartiles of daily alcohol intake (Table 4), we found a progressive increase in the prevalence of hypertriacylglyceridemia with higher consumption in women (<3.80 g/day: 7.5%, *n* = 4 versus ≥28.41 g/day: 25%, *n* = 10; *χ* ^2^ = 9.600; *p* = 0.04). In contrast, among men there was no clear trend regarding alcohol intake quartiles and MS components, although hypertriacylglyceridemia was the most closely linked factor for this categorization (*χ* ^2^ = 9.794, *p* = 0.04).

In consonance, evaluation of the association between the conglomerate classification and MS components revealed hypertriacylglyceridemia to show the greatest degree of association, progressively increasing in prevalence across groups (low intake: 13.8%, *n* = 22 versus high intake: 44.4%, *n* = 4; *χ* ^2^ = 10.980; *p* = 0.01). No other components showed a significant association with this categorization in females; and no link was found between these clusters and any MS components in males (Table 5).

### 3.3. Serum Triacylglycerides and Alcohol Consumption

**Figure 2:**
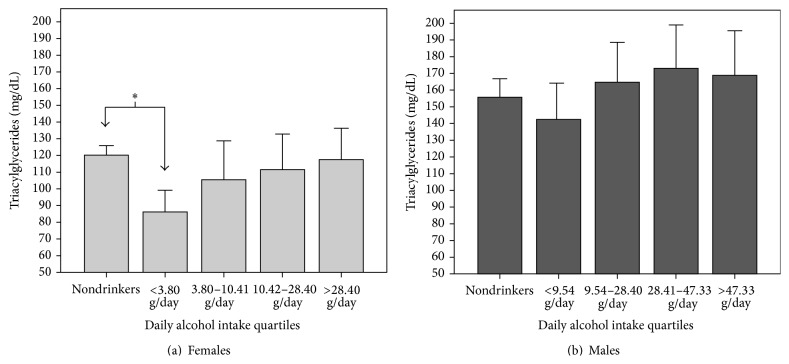
Serum triacylglyceride concentration by gender and daily alcohol intake quartiles. Maracaibo, 2015. *∗* One-way ANOVA. Post hoc Tukey: *p* = 0.046.

Figure 2 illustrates the epidemiologic behavior of serum TAG levels by gender and daily alcohol intake quartiles. Women in the <3.8 g/day group had significantly lower TAG than nondrinkers (86.2 ± 43.5 mg/dL versus 120.1 ± 91.9 mg/dL, resp.; *p* = 0.046). No significant differences were found concerning TAG levels and daily alcohol intake quartiles in men.

**Figure 3:**
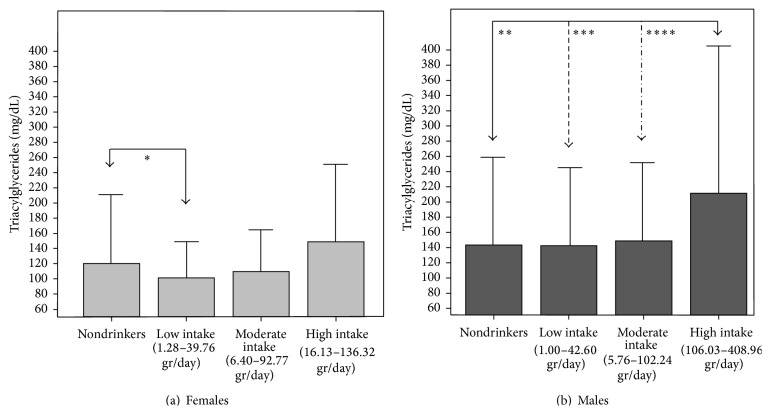
Serum triacylglyceride concentration by gender and drinking patter conglomerates. Maracaibo, 2015. One-way ANOVA. Post hoc Tukey: ^*∗*^ *p* = 0.053, ^*∗∗*^ *p* = 0.017, ^*∗∗∗*^ *p* = 0.016, ^*∗∗∗∗*^ *p* = 0.05.

On the other hand, Figure 3 depicts the behavior of serum TAG concentration by gender and drinking pattern clusters. Females in the low-intake conglomerate had lower TAG than nondrinkers, without statistical significance (101.2 ± 53.9 mg/dL versus 120.1 ± 91.99 mg/dL, resp.; *p* = 0.053). Among males, individuals in the high-intake conglomerate had significantly higher TAG (210.17 ± 192.58 mg/dL) than those in the low and moderate-intake groups.

### 3.4. Alcohol Consumption and Risk of Metabolic Syndrome and Its Components

Multivariate analysis of the relationship between drinking and MS and its components indicated hypertriacylglyceridemia to be the most tightly associated with this life style in females. Indeed, in this gender, analysis by daily alcohol intake quartiles (Table 6) showed subjects in the group with <3.80 g/day to have a lower risk of hypertriacylglyceridemia than nondrinkers (OR = 0.29, CI 95%: 0.09–086; *p* = 0.03). Likewise, analysis by clusters also demonstrated low-intake drinkers to have a lower risk of elevated TAG levels than nondrinkers (OR = 0.50, CI 95%: 0.29–0.84; *p* = 0.01), while those with higher consumption showed higher risk for MS and high TAG (Table 7).

Meanwhile, in males, multivariate analysis by daily alcohol intake quartiles (Table 8) found individuals in the 28.41–47.33 g/day category to have a greater risk of MS, hyperglycemia, elevated WC, high blood pressure, and high TAG, whereas those consuming ≥47.34 g/day had a reduced risk of low HDL-C (OR = 0.52, CI 95%: 0.34–0.79; *p* < 0.01). Finally, in men, analysis by conglomerates found high-intake drinkers to be associated with hyperglycemia (OR = 3.18; CI 95%: 1.25–8.14; *p* = 0.02) and moderate-intake drinkers to be associated with Low HDL-C and high blood pressure (Table 9).

## 4. Discussion

Psychobiologic life styles play an indisputable role in global well-being and are particularly prominent in the development of cardiometabolic alterations, with diet, physical activity, smoking, and alcohol consumption attracting vast scientific attention in recent decades [23]. Although elements such as physical inactivity and hypercaloric diets are regarded as largely ubiquitous westernized lifestyles propagated by mass globalization [24], drinking patterns tend to be dictated by population-specific geographic, sociodemographic, and cultural factors [1]. Therefore, it is important to analyze these characteristics in each population and their individual and/or accumulative relation to cardiometabolic disorders. Numerous studies have described the influence of alcohol consumption on the prevalence of MS, with a large part of these delineating a direct relationship between drinking and this condition [25, 26]. However, this is not a universal finding [13, 27], as evidenced in this study, even when utilizing two distinct classification systems for alcohol intake.

Initially, alcohol consumption was evaluated in quartiles according to sex but not taking into account the beverage consumed by the subjects. Later, a cluster analysis according to sex was calculated adding the daily quantity of alcohol consumption per beverage [19]. Interestingly, although quartile categorization yields evenly distributed groups, cluster analysis allows the delimitation of smaller-sized groups with alcohol intake much greater than that of habitual drinkers. This methodological heterogeneity varies according to daily consumption quantities which are influenced by several factors, including sociocultural norm, type of alcohol consumed, and dietary behavior. This discrepancy is important when analyzing nonsignificant results* a priori*.

We have ascertained a lower prevalence of MS in female subjects with low alcohol intake, in concordance with the findings of Lee et al. [28], who determined MS prevalence to be reduced among women with low or moderate consumption in their population. Furthermore, Wakabayashi [29] has described females with low alcohol intake to have lower MS prevalence than nondrinkers; and Freiberg et al. [30] have reported low-moderate drinkers to have lower MS prevalence (47% in females, 48% in males) in comparison to nondrinkers, independent of ethnic origin.

When individually studying clinical-metabolic alterations, we found hypertriacylglyceridemia to behave similarly to MS, again in the female gender. This trend echoes the results of the ATTICA study, where, after a 10-year follow-up, moderate alcohol consumption (1-2 drinks/day) was associated with decreased incidence of high TAG [31]. This phenomenon has been suggested to be related to the fact that low-intake drinkers predominantly consume wine, instead of beer and spirit drinks; thus, the bioflavonoids in this beverage may be responsible for this protective effect [32].

Multivariate analysis further supports this finding in women, with the impact of drinking confined to serum TAG concentration. Indeed, low alcohol consumption appeared to act as a protective factor when studied through daily intake quartiles, similar to the results of Clerc et al. [33], who attributed this finding to complex interplay between alcohol, carbohydrate and lipoprotein metabolism, insulin secretion, and energetic balance. Conversely, we observed that high intake acts as a risk factor for high TAG as well as for MS when evaluated through drinking pattern conglomerates. These results are in agreement with a report by Chen et al. [34], who determined consumption of ≥10 g/day to be associated with hypertriacylglyceridemia. Therefore, although our results differed depending on which classification was applied, both systems delineate a similar behavior for alcohol and serum TAG levels in women.

In contrast, in men, univariate analysis revealed a weak or absent degree of statistical association between drinking and MS or its components. Nonetheless, multivariate assessment yielded significant results concerning the group consuming 28.41–47.33 g/day (third quartile of daily intake), who displayed increased risk of MS. This concurs with observations in 19,000 Chinese subjects, although these were exclusive wine consumers [35]. In our study, individuals in this group also exhibited a greater risk of high blood pressure, as previously stated in a meta-analysis comprising 156 studies on the relationship between alcohol and this disorder [36]. Also in this group, drinking was strongly associated with hyperglycemia, almost doubling the risk of nondrinkers for this condition, resembling findings in an elderly Italian population. This effect has been proposed to be due to enhanced peripheral insulin sensitivity in response to moderate drinking, which is lost with greater quantities of alcohol [37].

Our finding of ≥47.34 gr/day intake or moderate intake by conglomerates acting as a protective factor for low HDL-C is consistent with reports from studies with differing methodology and drinking patterns: The Cooper Center Longitudinal Study, realized in 3,000 healthy American subjects, showed men with high consumption to have the highest HDL-C values (55.9 ± 11.3 mg/dL), while nondrinkers and low-intake drinkers displayed greater risk for this dyslipidemia [38]. Similarly, in a meta-analysis, Brien et al. [39] ascertained an ascending dose-response relationship between the quantity of alcohol consumed and HDL-C levels. Future research should explore whether the effect of alcohol on this lipoprotein influences cardiovascular outcomes, the antiatherogenic properties and functionality of these molecules in this scenario, and the potential impact of the daily drinking of these quantities of alcohol in other organ systems [40].

The limitations of this study include its cross-sectional design which hinders the possibility of determining causality relationships and the lack of nutritional data which might add another measure of influence over alcohol consumption frequency and quantity. Currently, this branch of research is being conducted as part of the MMSPS, which will render the necessary data to answer this important inquiry, especially in light of MS risk.

Finally, it is important to highlight that in our population the protective effect of alcohol against high TAG in women appears to occur with daily intake quantities equal to less than half the standard measures of the main alcoholic beverages in our locality. On the other hand, in men, the effects of alcohol on MS and its components are apparent with daily quantities equal to 4–6 beers, 3–5 spirit drinks, or 4–7 glasses of wine. These values should be utilized by clinicians as reference for educational efforts regarding the role of this substance in cardiometabolic health in our community. We also support evaluation by daily intake quartiles as the most easily applicable and reproducible method for quantitative assessment of this practice in our region, never forgetting to take into account those subjects with high alcohol intake.

## Figures and Tables

**Table 1:** Characteristics of general population by gender. Maracaibo, 2015.

	Females		Males		Total	
	*n* = 1172		*n* = 1058		*n* = 2230	
	*n*	%	*n*	%	*n*	%
Age groups (years)						
18–29	349	29.8	413	39.0	762	34.2
30–44	325	27.7	297	28.1	622	27.9
45–59	346	29.5	259	24.5	605	27.1
≥60	152	13.0	89	8.4	241	10.8
Ethnic groups						
Mixed	876	74.7	816	77.1	1692	75.9
Hispanic white	191	16.3	161	15.2	352	15.8
Afro-Venezuelan	30	2.6	36	3.4	66	3.0
Amerindian	62	5.3	44	4.2	106	4.8
Others	13	1.1	1	0.1	14	0.6
Alcohol intake quartiles						
Nondrinkers	976	83.3	582	55.0		
Quartile 1	53	4.5	119	11.2	—	—
Quartile 2	45	3.8	145	13.7	—	—
Quartile 3	58	4.9	89	8.4	—	—
Quartile 4	40	3.4	123	11.6	—	—
Alcohol intake pattern (conglomerates)						
Nondrinkers	976	83.3	580	54.8	—	—
Low intake	160	13.7	328	31.0	—	—
Moderate intake	27	2.3	124	11.7	—	—
High intake	9	0.8	26	2.5	—	—
Metabolic Syndrome^†^	474	40.4	472	44.6	946	42.4
High blood pressure^†^	410	35.0	456	43.1	866	38.8
Hyperglycemia^†^	301	25.7	322	30.4	623	27.9
Low HDL-C^†^	752	64.2	536	50.7	1288	57.8
High triacylglycerides^†^	269	23.0	347	32.8	616	27.6
Abdominal obesity^†^	926	79.0	749	70.8	1675	75.1
Body Mass Index classification (kg/m^2^)						
<24.9	420	35,8	275	26,0	695	31,2
25.0–29.9	371	31,7	415	39,2	786	35,2
≥30.0	381	32,5	368	34,8	749	33,6

^†^According IDF/AHA/NHLBI/WHF/IAS/IASO 2009 criteria.

**Table 2:** Prevalence of Metabolic Syndrome by gender and daily alcohol intake quartiles. Maracaibo, 2015.

	With Metabolic Syndrome		*χ* ^2^ (*p*)^**∗**^
	*n*	%	
Females			**9.332 (0.053)**
Nondrinkers	407	41.7	
<3.80 g/day	12	22.6	
3.80–10.41 g/day	14	31.1	
10.42–28.40 g/day	24	41.4	
≥28.41 g/day	17	42.5	
Males			**6.763 (0.149)**
Nondrinkers	247	42.4	
<9.54 g/day	49	41.2	
9.54–28.40 g/day	71	49.0	
28.41–47.33 g/day	49	55.1	
≥47.34 g/day	56	45.5	

^*∗*^Pearson's Chi-squared test.

**Table 3:** Prevalence of Metabolic Syndrome by gender and drinking pattern conglomerates. Maracaibo, 2015.

	With Metabolic Syndrome		*χ* ^2^ (*p*)^*∗*^
	*n*	%	
Females (gr/day)			**6.372 (0.095)**
Nondrinkers	407	41.7	
Low intake (1.28–39.76)	51	31.9	
Moderate intake (6.40–92.77)	11	40.7	
High intake (16.13–136.32)	5	55.6	
Males (gr/day)			**3.166 (0.367)**
Nondrinkers	246	42.4	
Low intake (1.00–42.60)	151	46.0	
Moderate intake (5.76–102.24)	62	50.0	
High intake (106.03–408.96)	13	50.0	

^*∗*^Pearson's Chi-squared test.

**Table 4:** Prevalence of Metabolic Syndrome components by gender and daily alcohol intake quartiles. Maracaibo, 2015.

	Hyperglycemia		Low HDL-C		Elevated waist circumference		High blood pressure		High TAG	
	*n*	%	*n*	%	*n*	%	*n*	%	*n*	%
Females										
Nondrinkers	253	25.9	635	65.1	769	78.8	354	36.3	236	24.2
<3.80 g/day	11	20.8	28	52.8	37	69.8	14	26.4	4	7.5
3.80–10.41 g/day	13	28.9	29	64.4	37	82.2	8	17.8	7	15.6
10.42–28.40 g/day	14	24.1	35	60.3	50	86.2	20	34.5	12	20.7
≥28.41 g/day	10	25.0	25	62.5	33	82.5	14	35.0	10	25.0
Males										
Nondrinkers	165	28.4	305	52.4	402	69.1	244	41.9	176	30.2
<9.54 g/day	38	31.9	58	48.7	87	73.1	49	41.2	33	27.7
9.54–28.40 g/day	41	28.3	76	52.4	108	74.5	64	44.1	56	38.6
28.41–47.33 g/day	37	41.6	46	51.7	70	78.7	46	51.7	38	42.7
>47.33 g/day	41	33.3	51	41.5	82	66.7	53	43.1	44	35.8

HDL-C: High-Density Lipoprotein-Cholesterol. TAG: triacylglycerides.

Pearson's Chi-squared test (*p*):

*Females*: hyperglycemia: *χ* ^2^ (*p*) = 1.028 (0.90); low HDL-C: *χ* ^2^ (*p*) = 3.720 (0.44); elevated waist circumference: *χ* ^2^ (*p*) = 5.118 (0.27); high blood pressure: *χ* ^2^ (*p*) = 8.285 (0.08); *high TAG*: *χ* ^2^ (*p*) = 9.600 (0.04).

*Males*: hyperglycemia: *χ* ^2^ (*p*) = 7.343 (0.11); low HDL-C: *χ* ^2^ (*p*) = 5.263 (0.26); elevated waist circumference: *χ* ^2^ (*p*) = 5.768 (0.21); high blood pressure: *χ* ^2^ (*p*) = 3.246 (0.51); *high TAG*: *χ* ^2^ (*p*) = 9.794 (0.04).

**Table 5:** Prevalence of Metabolic Syndrome components by gender and drinking pattern conglomerates. Maracaibo, 2015.

	Hyperglycemia		Low HDL-C		Elevated waist circumference		High blood pressure		High TAG	
	*n*	%	*n*	%	*n*	%	*n*	%	*n*	%
Females (gr/day)										
Nondrinkers	253	25.9	635	65.1	769	78.8	354	36.3	236	24.2
Low intake (1.28–39.76)	40	25.0	93	58.1	125	78.1	44	27.5	22	13.8
Moderate intake (6.40–92.77)	7	25.9	19	70.4	24	88.9	9	33.3	7	25.9
High intake (16.13–136.32)	1	11.1	5	55.6	8	88.9	3	33.3	4	44.4
Males (gr/day)										
Nondrinkers	165	28.4	304	52.4	401	69.1	242	41.7	176	30.3
Low intake (1.00–42.60)	102	31.1	165	50.3	243	74.1	143	43.6	113	34.5
Moderate intake (5.76–102.24)	42	33.9	56	45.2	84	67.7	60	48.4	46	37.1
High intake (106.03–408.96)	13	50.0	11	42.3	21	80.8	11	42.3	12	46.2

HDL-C: High-Density Lipoprotein-Cholesterol. TAG: triacylglycerides.

Pearson's Chi-squared test (*p*):

*Females*: hyperglycemia: *χ* ^2^ (*p*) = 1.070 (0.78); low HDL-C: *χ* ^2^ (*p*) = 3.622 (0.30); elevated waist circumference: *χ* ^2^ (*p*) = 2.222 (0.52); high blood pressure: *χ* ^2^ (*p*) = 4.693 (0.19); *high TAG*: *χ* ^2^ (*p*) = 10.980 (0.01).

*Males*: hyperglycemia: *χ* ^2^ (*p*) = 6.542 (0.08); low HDL-C: *χ* ^2^ (*p*) = 2.956 (0.39); elevated waist circumference: *χ* ^2^ (*p*) = 4.298 (0.23); high blood pressure: *χ* ^2^ (*p*) = 1.901 (0.59); high TAG: *χ* ^2^ (*p*) = 5.13 (0.16).

**Table 6:** Adjusted odds ratios for Metabolic Syndrome and its components by daily alcohol intake quartiles in females. Maracaibo, 2015.

	Metabolic Syndrome	High fasting glucose	Low HDL-C	High waist circumference	High blood pressure	High triacylglycerides
	OR (95% CI); *p*	OR (95% CI); *p*	OR (95% CI); *p*	OR (95% CI); *p*	OR (95% CI); *p*	OR (95% CI); *p*
Nondrinkers	1.00	1.00	1.00	1.00	1.00	1.00
<3.80 g/day	0.53 (0.25–1.12); 0.09	1.01 (0.48–2.14); 0.98	0.73 (0.41–1.32); 0.30	0.76 (0.36–1.61); 0.47	1.12 (0.54–2.34); 0.76	**0.29 (0.09–0.86)**; **0.03**
3.80–10.41 g/day	1.08 (0.51–2.28); 0.83	1.67 (0.79–3.50); 0.18	1.08 (0.57–2.07); 0.81	1.82 (0.75–4.39); 0.19	0.61 (0.25–1.50); 0.28	0.79 (0.32–1.93); 0.60
10.42–28.40 g/day	0.86 (0.45–1.63); 0.64	0.94 (0.48–1.86); 0.86	0.69 (0.39–1.24); 0.22	1.05 (0.45–2.46); 0.92	1.08 (0.55–2.09); 0.83	0.65 (0.31–1.38); 0.26
≥28.41 g/day	1.33 (0.59–2.94); 0.49	1.06 (0.47–2.36); 0.89	0.83 (0.41–1.67); 0.60	1.25 (0.49–3.23); 0.64	1.53 (0.64–3.63); 0.34	1.12 (0.48–2.58); 0.79

HDL-C: High-Density Lipoprotein-Cholesterol.

Models adjusted for age groups, ethnic groups, occupational status, educational status, socioeconomic status, family history of hypertension and diabetes, tobacco use, four domains of physical activity, and daily alcohol intake quartiles.

**Table 7:** Adjusted odds ratios for Metabolic Syndrome and its components by drinking pattern conglomerates in females. Maracaibo, 2015.

(gr/day)	Metabolic Syndrome	High fasting glucose	Low HDL-C	High waist circumference	High blood pressure	High triacylglycerides
	OR (95% CI); *p*	OR (95% CI); *p*	OR (95% CI); *p*	OR (95% CI); *p*	OR (95% CI); *p*	OR (95% CI); *p*
Nondrinkers	1.00	1.00	1.00	1.00	1.00	1.00
Low intake (1.28–39.76)	0.77 (0.50–1.18); 0.23	1.17 (0.76–1.80); 0.48	0.76 (0.53–1.09); 0.14	1.00 (0.61–1.61); 0.98	1.00 (0.64–1.57); 0.99	**0.50 (0.29–0.84)**; **0.01**
Moderate intake (6.40–92.77)	0.97 (0.39–2.42); 0.94	1.08 (0.42–2.78); 0.87	1.40 (0.58–3.35); 0.45	1.69 (0.45–6.32); 0.44	1.07 (0.40–2.86); 0.89	1.03 (0.39–2.75); 0.95
High intake (16.13–136.32)	**4.96 (1.09–22.56)**; **0.04**	0.49 (0.05–4.43); 0.52	0.59 (0.15–2.36); 0.46	4.24 (0.47–38.14); 0.19	1.85 (0.33–10.47); 0.49	**5.87 (1.31–26.23)**; **0.02**

HDL-C: High-Density Lipoprotein-Cholesterol.

Models adjusted for age groups, ethnic groups, occupational status, educational status, socioeconomic status, family history of hypertension and diabetes, tobacco use, four domains of physical activity, and drinking pattern conglomerates.

**Table 8:** Adjusted odds ratios for Metabolic Syndrome and its components by daily alcohol intake quartiles in males. Maracaibo, 2015.

	Metabolic Syndrome	High fasting glucose	Low HDL-C	High waist circumference	High blood pressure	High triacylglycerides
	OR (95% CI); *p*	OR (95% CI); *p*	OR (95% CI); *p*	OR (95% CI); *p*	OR (95% CI); *p*	OR (95% CI); *p*
Nondrinkers	1.00	1.00	1.00	1.00	1.00	1.00
<9.54 g/day	0.94 (0.59–1.50); 0.79	1.35 (0.84–2.18); 0.21	0.83 (0.55–1.26); 0.39	1.28 (0.76–2.16); 0.35	1.08 (0.58–1.72); 0.76	0.79 (0.49–1.28); 0.34
9.54–28.40 g/day	1.26 (0.81–1.95); 0.31	0.91 (0.58–1.43); 0.68	0.88 (0.59–1.31); 0.53	1.32 (0.81–2.16); 0.27	1.15 (0.74–1.79); 0.53	1.32 (0.86–2.01); 0.20
28.41–47.33 g/day	**1.86 (1.11–3.12)**; **0.02**	**1.99 (1.20–3.33)**; **<0.01**	0.89 (0.56–1.43); 0.64	**2.13 (1.14–3.98)**; **0.02**	**1.95 (1.16–3.29)**; **0.01**	**1.63 (1.00–2.68)**; **0.05**
≥47.34 g/day	1.23 (0.77–1.97); 0.39	1.45 (0.90–2.34); 0.13	**0.52 (0.34–0.79)**; **<0.01**	1.05 (0.63–1.76); 0.84	1.42 (0.88–2.28); 0.15	1.15 (0.72–1.83); 0.56

HDL-C: High-Density Lipoprotein-Cholesterol.

Models adjusted for age groups, ethnic groups, occupational status, educational status, socioeconomic status, family history of hypertension and diabetes, tobacco use, four domains of physical activity, and daily alcohol intake quartiles.

**Table 9:** Adjusted odds ratios for Metabolic Syndrome and its components by drinking pattern conglomerates in males. Maracaibo, 2015.

gr/day	Metabolic Syndrome	High fasting glucose	Low HDL-C	High waist circumference	High blood pressure	High triacylglycerides
	OR (95% CI); *p*	OR (95% CI); *p*	OR (95% CI); *p*	OR (95% CI); *p*	OR (95% CI); *p*	OR (95% CI); *p*
Nondrinkers	1.00	1.00	1.00	1.00	1.00	1.00
Low intake (1.00–42.60)	1.18 (0.85–1.63); 0.32	1.19 (0.85–1.66); 0.30	0.85 (0.64–1.14); 0.29	1.42 (0.99–2.03); 0.06	1.23 (0.89–1.71); 0.21	1.11 (0.81–1.53); 0.52
Moderate intake (5.76–102.24)	1.46 (0.92–2.32); 0.11	1.41 (0.88–2.25); 0.15	**0.61 (0.40–0.94)**; **0.02**	0.97 (0.58–1.61); 0.90	**1.83 (1.15–2.92)**; **0.01**	1.15 (0.73–1.81); 0.55
High intake (106.03–408.96)	1.33 (0.51–3.44); 0.56	**3.18 (1.25–8.14)**; **0.02**	0.58 (0.25–1.38); 0.22	3.33 (0.92–12.06); 0.07	1.02 (0.39–2.68); 0.97	1.98 (0.79–4.89); 0.14

HDL-C: High-Density Lipoprotein-Cholesterol.

Models adjusted for age groups, ethnic groups, occupational status, educational status, socioeconomic status, family history of hypertension and diabetes, tobacco use, four domains of physical activity, and drinking pattern conglomerates.
